# Brain Aging Mediating Heart Imaging‐Derived Phenotypes and Mental and Nervous System Disorders

**DOI:** 10.1111/acel.70499

**Published:** 2026-04-23

**Authors:** Zairen Zhou, Wenjing Su, Yuna Li, Bei Zhang, Fang Lan, Dawei Lin, Jin‐Tai Yu, Jianfeng Feng, Yifei Jin, Peng Ren, Wei Cheng

**Affiliations:** ^1^ Institute of Science and Technology for Brain‐Inspired Intelligence, Department of Neurology, Huashan Hospital, State Key Laboratory of Brain Function and Disorders and MOE Frontiers Center for Brain Science Fudan University Shanghai China; ^2^ Department of Radiology Beijing Tiantan Hospital, Capital Medical University Beijing China; ^3^ Brain Health Institute, National Center for Mental Disorders, Shanghai Mental Health Center Shanghai Jiao Tong University School of Medicine and School of Psychology Shanghai China; ^4^ Department of Cardiology Zhongshan Hospital, Fudan University Shanghai China; ^5^ Key Laboratory of Computational Neuroscience and Brain Inspired Intelligence (Fudan University), Ministry of Education Shanghai China; ^6^ School of Data Science, Fudan University Shanghai China; ^7^ Department of Computer Science University of Warwick Coventry UK; ^8^ Fudan ISTBI—ZJNU Algorithm Centre for Brain‐Inspired Intelligence Zhejiang Normal University Jinhua China

**Keywords:** brain age, brain disorder, heart, MRI

## Abstract

Mental and nervous system disorders often co‐occur with cardiovascular diseases in aging populations, yet the biological relationships underlying these associations remain incompletely understood. Using heart and brain imaging data from 33,573 UK Biobank (UKB) participants, we developed a brain age prediction model to estimate the brain age gap (BAG), an imaging‐based marker of brain aging. We then examined BAG as a mediator between 82 cardiac imaging‐derived phenotypes (IDPs) and 11 disorders. Sixty‐one cardiac IDPs, particularly those related to the atria and left ventricle, were significantly associated with BAG, with several also related to mental and nervous system disorders. Mediation analyses revealed that BAG significantly mediated 18 associations between heart and substance abuse, mood, and neurotic disorders. Furthermore, we observed a total of 49 significant associations, where lifestyle factors, including smoking and physical activity, were related to heart‐brain aging‐disorder relationships. These findings highlight brain aging as a potential pathway linking cardiovascular health to diverse brain disorders in aging populations.

## Graphical abstract

Using heart and brain imaging from 33,573 UK Biobank participants, we establish a clear association between brain aging and cardiac structural changes. In addition, we uncover several potentially modifiable lifestyle and environmental factors that contribute to mental and nervous system disorders, mediated through alterations in cardiac structure and accelerated brain aging.

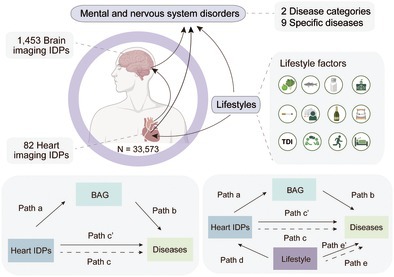

## 1 Introduction

Mental and nervous system disorders as well as cardiovascular diseases are highly prevalent in the aging population (Luo et al. 2025; Hu et al. 2022; Canever et al. 2024; Martin et al. 2025), and comorbidity between the two has been frequently reported (Bergstedt et al. 2024; Tahsili‐Fahadan and Geocadin 2017; Chen et al. 2024). Emerging evidence indicates that individuals with cardiovascular disease are at higher risk of developing mental and nervous system disorders (Shen et al. 2022; Zhang et al. 2024). For example, a recent systematic review and meta‐analysis reported that patients with heart failure have a 60% increased risk of developing dementia (Wolters et al. 2018), and epidemiological studies estimate that the 20%–50% of individuals with heart failure experience comorbid anxiety and depression (Celano et al. 2018; Tsabedze et al. 2021). These comorbidities have been partly attributed to mechanisms such as inflammation and cerebral microvascular dysfunction (Wolters et al. 2018; Celano et al. 2018; Justin et al. 2013). However, these mechanisms are unlikely to fully account for the complex associations between cardiac health and mental and nervous system disorders.

Emerging research has proposed the heart‐brain axis as a conceptual framework to understand how the cardiovascular system influences brain physiology through neural and neuroendocrine pathways (Valenza et al. 2025; Hu et al. 2023). The autonomic nervous system modulates cerebral blood flow to meet the brain's metabolic demands (ter Laan et al. 2013; Willie et al. 2014; Koep et al. 2022) by regulating heart rate and blood pressure (Florea and Cohn 2014; Gibbons 2019). While neurovascular coupling further ensures a continuous supply of oxygen and nutrients to the brain, facilitated by the cardiac output (Blinder et al. 2013; Phillips et al. 2016; Iadecola 2017; Stackhouse and Mishra 2021). As a clinically validated technology, Magnetic resonance imaging (MRI) has been extensively utilized to characterize the structure and function of both the heart and brain. Existing evidence has demonstrated that heart and brain imaging traits are interconnected not only phenotypically but also genetically (Zhao et al. 2023; McCracken et al. 2022; Jaggi et al. 2024). However, most of these phenotypic and genetic studies focus on the regional‐level associations between the heart and brain, neglecting the fact that the brain is interconnected as a whole. The investigation of the connections between the heart and the whole brain could give more biologically plausible pathways for studying brain disorders.

Brain aging, a comprehensive estimation of the whole brain health status, is increasingly recognized as a crucial biological process that contributes to cognitive decline and neuropsychiatric disorders in later life. The neuroimaging‐based concept of ‘brain age gap’ (Tanveer et al. 2023; Tian et al. 2023), which quantifies whether an individual exhibited accelerated or delayed brain aging compared to their normal peers, has been associated with increased risk for multiple mental and nervous system disorders (Solh and Cevher 2025; Hou et al. 2019). Moreover, cardiac function and vascular health, such as systolic and diastolic function, have also been linked to brain aging processes (Jefferson 2010; Ovsenik et al. 2021), including greater gray matter atrophy and white matter hyperintensity (Yaqub et al. 2025). Furthermore, a recent multi‐organ predictive model reported that heart aging showed the strongest associations with brain aging across multiple physiological systems (Tian et al. 2023). These findings suggest that brain aging may serve as a shared pathway linking cardiovascular health to mental and nervous system disorders, especially in the aging population. However, the role of neuroimaging‐based brain aging in mediating this pathway remains underexplored. Integrating heart IDPs and BAG may therefore offer a promising approach to investigate how cardiac health is linked to overall brain structural and functional changes and the risk of mental and nervous system disorders.

In this study, utilizing heart and brain imaging data from the UKB cohort, we aimed to investigate whether brain aging could mediate the association between heart IDPs and mental and nervous system disorders. Specifically, we (1) developed a brain biological age prediction model to calculate BAG, (2) examined the associations between heart IDPs and BAG, as well as heart IDPs and disease outcomes, (3) investigated the mediating role of BAG in the relationship between heart IDPs and mental/nervous system disorders, and (4) identified potential modifiable environmental and lifestyle factors underlying the heart–brain aging–disease relationships. By integrating large‐scale heart and brain imaging data with health and lifestyle information, this study provides a novel mechanistic framework in which brain aging serves as a plausible mediated pathway linking cardiac function to brain disorders. Our work offers a new perspective on the heart‐brain axis and may provide early identification and lifestyle‐targeted preventative strategies in the aging populations.

## 2 Results

### 2.1 Study Population and Data Overview

**FIGURE 1:**
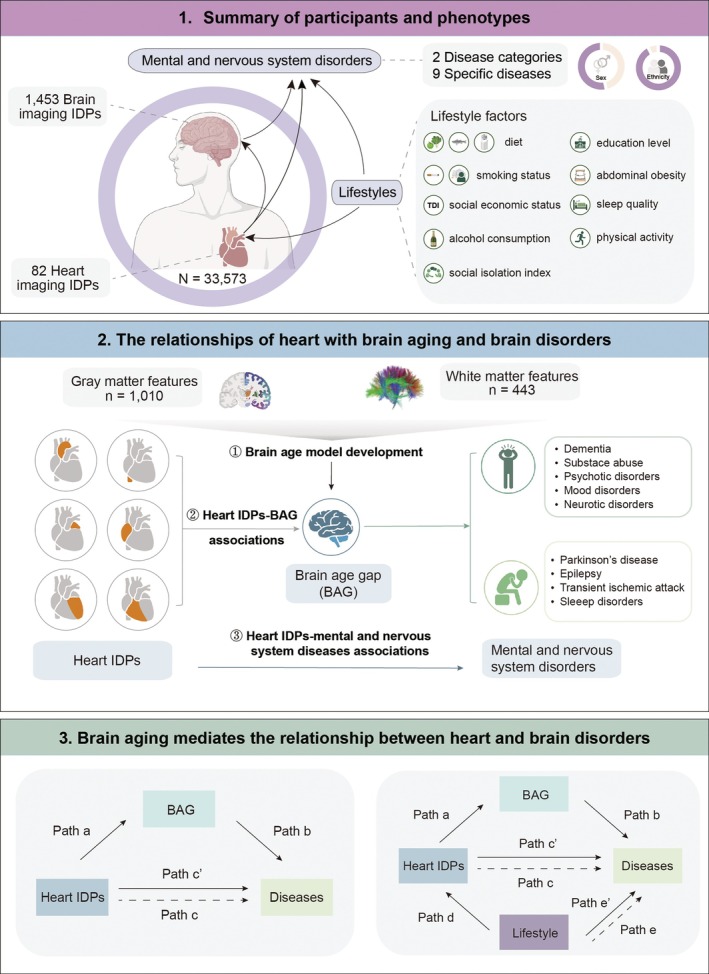
Overview of study workflow. Utilizing data from 33,573 participants with heart and brain imaging in the UK Biobank cohort, we investigated whether brain aging mediates the relationship between heart IDPs and mental and nervous system disorders. We first developed a brain biological age prediction model using LASSO regression to compute the BAG. Next, we used GLMs to assess associations between heart IDPs and BAG, as well as between heart IDPs and disease outcomes. Cox proportional hazards models were applied to examine incident disease risks. We then performed SEM to test the mediating role of BAG in these associations. Finally, we used SEM to identify modifiable environmental and lifestyle factors that may be associated with the heart–brain aging–disease pathway. BAG, brain age gap; GLM, generalized linear models; IDPs, imaging‐derived phenotypes; SEM, structural equation modeling.

We utilized 82 heart IDPs derived from cardiovascular short‐axis, long‐axis, and aortic cine MRI data and 1453 brain IDPs derived from T1‐weighted and diffusion‐weighted brain imaging data from the UKB cohort (Methods). The heart IDPs included global measures of two aortic sections, the ascending aorta (AAo) and descending aorta (DAo), and four cardiac chambers, the left ventricle (LV), right ventricle (RV), left atrium (LA), and right atrium (RA). A total of 33,573 participants (aged 63.5 ± 7.5 years, 53.3% female, 97.2% white ethnicity) were included in this study. Detailed demographic characteristics are presented in Table 1. A total of 11 mental and nervous system disorders were analyzed in UKB, which were divided into two broad categories (mental and behavioral disorders and nervous system disorders), with the remaining nine being non‐exhaustive representative diseases from these two categories. From the first broad category, mental and behavioral disorders, five disorders were further analyzed: dementia, substance abuse, psychotic disorders, mood disorders, and neurotic disorders. From the second broad category, nervous system disorders, four disorders were analyzed: Parkinson's disease, epilepsy, transient ischemic attacks, and sleep disorders. Sample sizes for each disease are summarized in Table S1. Nine groups of environmental and lifestyle factors, comprising 14 items, were retrieved in the study (Methods). Figure 1 provides an overview of the study.

**TABLE 1:** Demographic characteristics of participants.

Variables	Total *N* = 33,573
Age (years; mean ± SD)	63.5 ± 7.5
Sex (female, %)	17,895 (53.3%)
Ethnicity (white, %)	32,634 (97.2%)
TDI (mean ± SD)	−1.92 ± 2.7
BMI (mean ± SD)	26.4 ± 4.34
eTIV (mean ± SD)	1,548,550 ± 151,122
Scanning site (%)	
Cheadle	20,809 (62.0%)
Reading	4018 (12.0%)
Newcastle	8746 (26.1%)
Smoking status (%)	
Never	21,132 (62.9%)
Previous	11,289 (33.6%)
Current	1152 (3.43%)
Drinking status (%)	
Never	1067 (3.18%)
Previous	1102 (3.28%)
Current	31,404 (93.5%)
Mental and behavioral disorders (%)	
Prevalent	6112 (18.2%)
Incident	2744 (8.2%)
Healthy	24,717 (73.6%)
Nervous system disorders (%)	
Prevalent	5315 (15.8%)
Incident	3065 (9.1%)
Healthy	25,193 (75.1%)

Abbreviations: BMI, body mass index; eTIV, estimated total intracranial volume; SD, standard deviation; TDI, Townsend deprivation index.

### 2.2 Brain Age Models and Associations of Heart IDPs With BAG

**FIGURE 2:**
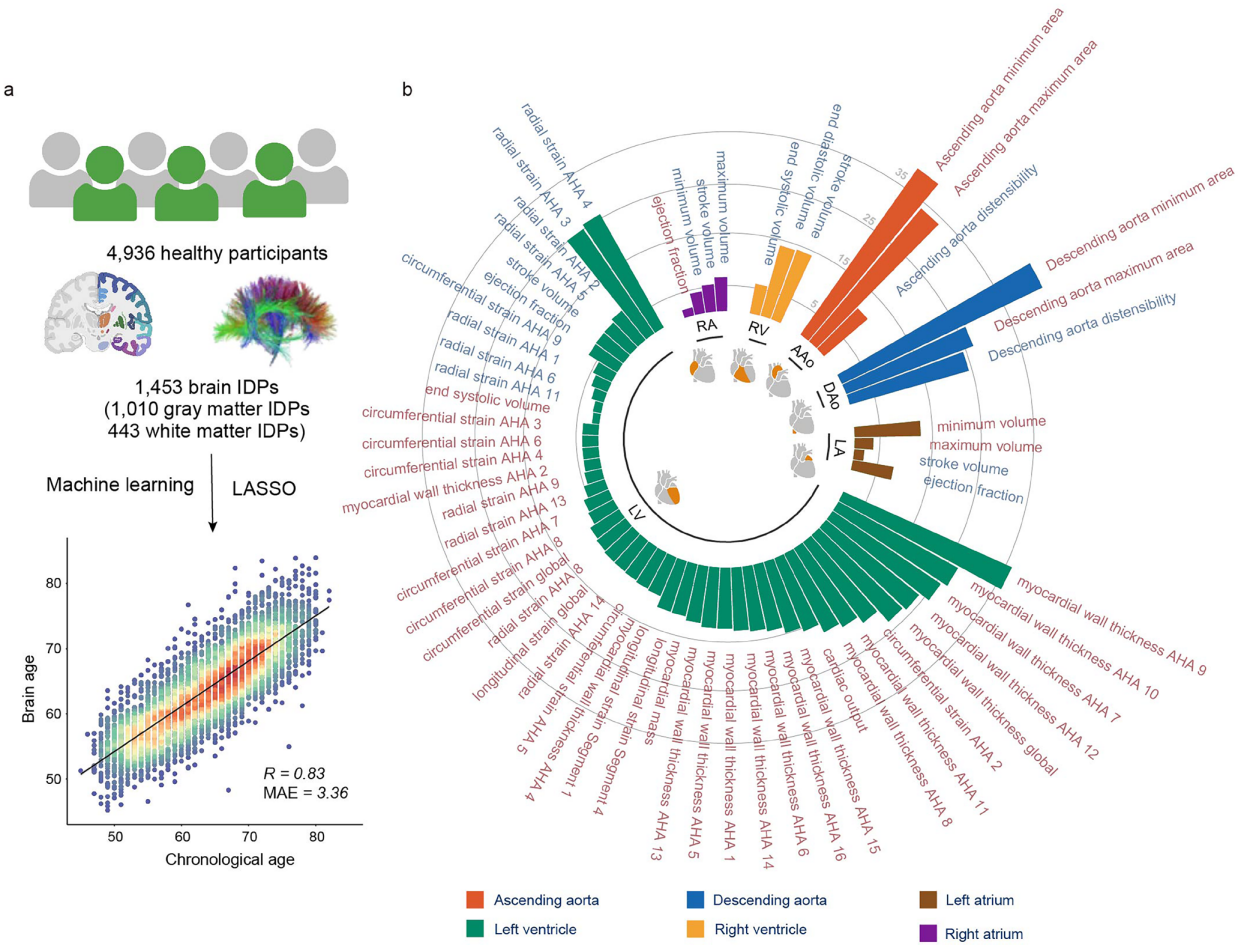
Brain biological age prediction model and associations between heart health and brain aging. (a) Schematic of brain age gap estimation. A brain age prediction model was developed using structural brain imaging data from 4936 healthy UK Biobank participants. A LASSO regression model was trained on brain IDPs to predict biological age. Model performance was evaluated using 10‐fold cross‐validation, achieving a Pearson's correlation of *R* = 0.83 and a MAE of 3.36 years between predicted biological and chronological age. The BAG was defined as predicted minus chronological age, with positive values indicating accelerated brain aging. (b) 61 significant associations between heart IDPs and BAG, identified using a generalized linear model. Each bar represents the standardized regression coefficient (*β*) for one heart IDP, with bar length indicating the magnitude of the association. Label colors represent directionality: red for positive associations (higher IDP values associated with greater BAG), and blue for negative associations (higher IDP values associated with lower BAG). The FDR is applied across all possible heart IDPs‐BAG pairs. Only those associations with FDR‐corrected *p* < 0.05 are considered significant, adjusted for chronological age, sex, smoking and drinking status, BMI, and Townsend index. BAG, brain age gap; BMI, body mass index; FDR, false discovery rate; IDPs, imaging‐derived phenotypes; MAE, mean absolute error.

To more comprehensively assess the status of brain aging, we identified substantial heterogeneity in brain IDPs among individuals of the same chronological age and provided unsupervised evidence of latent structure, supporting the motivation for modeling biological rather than chronological age (Supporting Information Methods, Figures S1–S3). To estimate the biological age of the brain, we trained a LASSO regression model using 1453 brain IDPs from 4936 healthy participants. The model demonstrated high predictive accuracy, with a Pearson's correlation coefficient of *R* = 0.83 and a mean absolute error (MAE) of 3.36 years between the predicted age and chronological age in the test sets (Figure 2a). When evaluated in a fold‐wise manner, the model demonstrated consistent performance across the 10 cross‐validation folds, with a mean MAE of 3.36 ± 0.14 years, a mean correlation of *r* = 0.83 ± 0.02, and a mean *R* ^2^ of 0.55 ± 0.05 (Table S2). The final LASSO model retained 551 imaging‐derived phenotypes with non‐zero coefficients.

Before bias correction, BAG showed a substantial negative correlation with chronological age (*r* = −0.55 both in healthy sample and full sample), consistent with known regression dilution effects in brain age models. After applying the correction procedure, the correlation was markedly reduced both in the healthy cross‐validation sample (*r* = −0.07) and in the full cohort (*r* = −0.05), indicating effective removal of age‐related bias (Figure S4).

In sensitivity analyses using alternative modeling approaches, both Support Vector Regression (SVR) and Random Forest (RF) exhibited inferior predictive performance compared with the primary LASSO model, characterized by larger mean absolute errors (MAE) and lower correlations with chronological age (SVR: MAE = 3.75, *r* = 0.79, *R* ^2^ = 0.62; RF: MAE = 4.25, *r* = 0.72, *R* ^2^ = 0.51). Detailed fold‐wise results are provided in Table S3. We next examined the relationship between heart IDPs and BAG using a generalized linear model (GLM). A total of 61 out of 82 heart IDPs showed significant associations with BAG after FDR correction (*p*_FDR_ < 0.05; Figure 2b). After hierarchical FDR correction, all the previously significant associations remained statistically significant. Significant results from the GLM regression models are summarized in Table S4. Across all the heart IDPs, the strongest positive and negative associations with BAG were both observed among aorta‐related IDPs. Six aorta‐related IDPs showed significant associations with BAG, including four positive (*β* = 0.355 to 0.466, *p*_FDR_ = 5.28 × 10^−44^ to 4.07 × 10^−27^) and two negative associations (*β* = −0.298 to −0.202, *p*_FDR_ = 8.01 × 10^−25^ to 7.67 × 10^−12^). Among these, the minimum area of the DAo exhibited the strongest positive association with BAG (*β* = 0.466, *p*_FDR_ = 5.28 × 10^−44^), whereas DAo distensibility showed the strongest negative association (*β* = −0.298, *p*_FDR_ = 8.01 × 10^−25^).

For atrium‐related IDPs, eight IDPs were significantly associated with BAG, comprising three positive (*β* = 0.062 to 0.202, *p*_FDR_ = 1.47 × 10^−13^ to 0.031) and five negative (*β* = −0.155 to −0.072, *p*_FDR_ = 1.07 × 10^−8^ to 0.01) relationships. Specifically, LA minimum volume showed the strongest positive association with BAG (*β* = 0.202, *p*_FDR_ = 1.47 × 10^−13^), while RA maximum volume exhibited the strongest negative association (*β* = −0.155, *p*_FDR_ = 2.59 × 10^−7^).

Associations with BAG were also found across ventricle‐related IDPs, with 47 of 68 phenotypes reaching statistical significance. Within LV IDPs, 34 were positively associated with BAG (e.g., myocardial wall thickness AHA 9, *β* = 0.071 to 0.420, *p*_FDR_ = 1.25 × 10^−36^ to 0.041), and 10 were negatively associated with BAG (e.g., LV radial strain AHA 4, *β* = −0.295 to −0.062, *p*_FDR_ = 5.47 × 10^−26^ to 0.029). For RV IDPs, lower end diastolic volume, stroke volume, and end systolic volume were correlated with increased BAG (*β* = −0.294 to −0.176, *p*_FDR_ = 7.32 × 10^−15^ to 1.63 × 10^−6^).

### 2.3 Associations of Heart IDPs With Mental and Nervous System Disorders

We examined associations between 82 heart IDPs and 11 mental and nervous system disorders. GLM regression models were used to assess the prevalence of pre‐existing conditions, while Cox proportional hazards models were applied to evaluate the associations with the incidence of disease.

**FIGURE 3:**
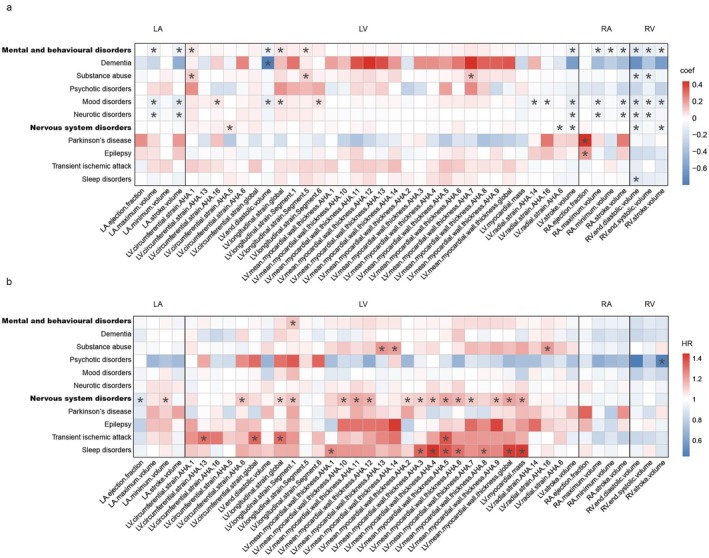
Associations between heart IDPs and prevalent and incident mental and nervous system diseases. (a) Heatmap demonstrating associations between heart IDPs (*x* axis) and 11 prevalent mental and nervous system diseases (*y* axis). The coefficient values are color‐coded, with warm color representing positive associations, and cool color representing negative associations. (b) Heatmap demonstrating the association between heart IDPs (*x* axis) and risk of 11 different mental and nervous system diseases (*y* axis). The HR values are color‐coded, with warm color representing positive associations between heart IDPs and disease risk, and cool color representing negative associations. In both panels, FDR is applied across all possible heart IDPs‐disease pairs. Associations with FDR‐corrected *p* < 0.05 are considered significant and marked with an asterisk. FDR, false discovery rate; HR, hazard ratio; IDPs, imaging‐derived phenotypes.

The GLM regression models identified 48 significant associations between heart IDPs and the prevalence of 11 brain disorders, which were divided into two broad categories (mental and behavioral disorders and nervous system disorders), with the remaining nine being non‐exhaustive representative diseases from these two categories. Results from the GLM regression models are presented in Table S5. Within the broad category of mental and behavioral disorders, four of the six heart IDP categories, excluding DAo and AAo IDPs, were significantly associated with disease prevalence (*β* = −0.129 to 0.052, *p*_FDR_ = 7.31 × 10^−10^ to 0.038) (Figure 3a). The strongest positive and negative associations were both found among ventricle‐related IDPs, represented by LV longitudinal strain global (*β* = 0.052, *p*_FDR_ = 0.004) and RV end diastolic volume (*β* = −0.129, *p*_FDR_ = 7.31 × 10^−10^), respectively. By contrast, atrium‐related IDPs showed exclusively negative correlations with the prevalence of mental and behavioral disorders, with RA stroke volume exhibiting the largest effect size (*β* = −0.087, *p*_FDR_ = 1.28 × 10^−7^). Among the specific mental and behavioral disorders included in this study, the strongest association was observed between LV IDP and dementia, whereby lower LV end diastolic volume was related to a higher prevalence of dementia (*β* = −0.797, *p*_FDR_ = 0.037). In addition, ventricle traits demonstrated significant associations with the prevalence of the broad category of nervous system disorders (*β* = −0.080 to 0.050, *p*_FDR_ = 3.31 × 10^−4^ to 0.038), with negative associations predominating. RV end diastolic volume exhibited the strongest negative correlation with disease prevalence (*β* = −0.080, *p*_FDR_ = 5.03 × 10^−4^). Among the specific nervous system disorders analyzed, the strongest positive association was found between RA ejection fraction and the prevalence of Parkinson's disease (*β* = 0.441, *p*_FDR_ = 4.86 × 10^−4^). In sensitivity analysis, 39 out of 48 significant associations between heart IDPs and the prevalence of brain disorders remained significant after hierarchical FDR correction (Table S5).

We further found 33 significant associations between heart IDPs and the risk of brain disorders using Cox proportional hazards models (Figure 3b). All significant results are detailed in Table S6. Since all heart IDPs were standardized prior to Cox modeling, the reported hazard ratios (HRs) represent the risk associated with a one standard deviation (SD) increase in each IDP. Among these, LV longitudinal strain Segment 1 was the only heart IDP that was associated with the broad category of mental and behavioral disorders (HR = 1.13, 95% confidence interval [CI] = 1.06–1.21, *p*_FDR_ = 0.006, SD = 6.92). When examining the specific mental and behavioral disorders, the strongest positive association was observed between LV mean myocardial wall thickness AHA 13 and the risk of substance abuse (HR = 1.23, 95% CI = 1.09–1.40, *p*_FDR_ = 0.013, SD = 0.76). Additionally, atrium and ventricle traits were significantly associated with the risks of the broad category of nervous system disorders (HR = 0.89 to 1.20, *p*_FDR_ = 2.63 × 10^−4^ to 0.042). The strongest association with disease risk was found among LV‐related IDPs, represented by LV mean myocardial wall thickness global (HR = 1.20, 95% CI = 1.10–1.31, *p*_FDR_ = 3.68 × 10^−4^, SD = 0.77). Among the specific nervous system disorders analyzed in our study, LV mean myocardial wall thickness AHA 4 exhibited the strongest association with the risk of sleep disorders (HR = 1.45, 95% CI = 1.23–1.70, *p*_FDR_ = 1.78 × 10^−4^, SD = 1.04). After hierarchical FDR correction, 21 of the previously significant associations between heart IDPs and incident brain disorders (*n* = 33) remained statistically significant (Table S6).

Sensitivity analyses excluding rare disorders (psychotic disorders and Parkinson's disease) yielded highly consistent results. Logistic regression coefficients were almost identical to the original estimates (*r* = 0.999; Table S7), and Cox model HRs showed strong concordance (*r* = 0.983; Table S8).

### 2.4 Brain Aging Mediates the Heart‐Mental and Nervous System Disorders Associations

Given the observed associations between heart IDPs, BAG, and mental and nervous system disorders, we hypothesized that BAG may serve as a mediator in these relationships. To test this, for significant heart‐disease associations, structural equation modeling (SEM) analyses were performed to identify significant mediating role of BAG (a × b, Figure 1), with heart IDPs as the exogenous variable, and disease phenotype as the outcome variable. Notably, given the observational nature of the data, the SEM results should be interpreted as reflecting associative relationships rather than causal correlations.

**FIGURE 4:**
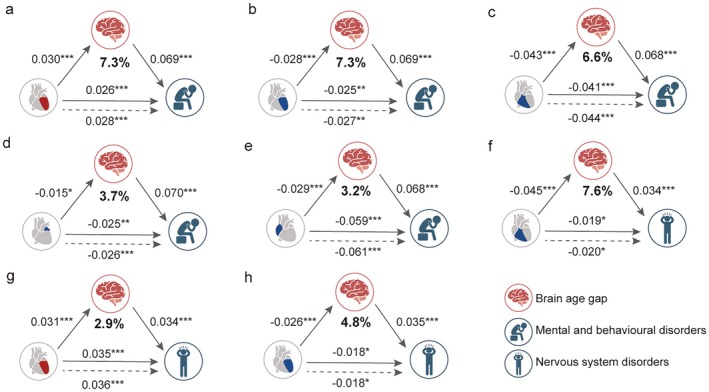
Significant mediating effects of BAG in the association between heart IDP categories and mental and nervous system diseases. Panels (a–h) illustrate significant pathways linking heart IDP categories (LV, RV, LA, RA), BAG, and mental and nervous system diseases. SEMs were fitted to evaluate the associations between heart IDP categories (exposure) and the diseases (outcome), including mental and behavioral disorders (a–e) and nervous system disorders (f–h), with BAG as the mediating factor. Color‐coded heart IDPs are displayed at their respective anatomic locations. Since BAG consistently exhibited positive associations with brain diseases, the red color indicates the positive associations between heart IDPs and BAG or diseases, while blue color represents the negative associations between heart IDPs and BAG or diseases. All models were estimated using a 1000‐iteration non‐parametric bootstrap approach. *FDR‐corrected *p* < 0.05; **FDR‐corrected *p* < 0.01; ***FDR‐corrected *p* < 0.001. BAG, brain age gap; FDR, false discovery rate; IDPs, imaging‐derived phenotypes; LA, left atrium; LV, left ventricle; RA, right atrium; RV, right ventricle; SEMs, structural equation modelings.

The SEM analysis revealed five statistically significant mediating effects of BAG in the association between four heart IDP categories (LA, LV, RA, and RV) and the broad category of mental and behavioral disorders (*p*_FDR_ < 0.05, Figure 4). All results are presented in Table S9. Among these, the largest mediation proportion was observed in the positive association between LV IDPs and mental and behavioral disorders, in which BAG accounted for 7.34% of the total mediating effect (indirect effect: *β* = 0.002, *p*_FDR_ = 1.16 × 10^−5^; Figure 4a). Additionally, BAG also mediated the negative association between ventricle‐related IDPs and the disease prevalence (proportion = 6.61% to 7.32%, indirect effect: *β* = −0.003 to −0.002, *p*_FDR_ = 2.03 × 10^−7^ to 4.61 × 10^−5^; Figure 4b,c). By contrast, BAG only exhibited mediating effects within the negative association between atrium‐related IDPs and the prevalence of the broad category of mental and behavioral disorders (proportion = 3.18% to 3.70%, indirect effect: *β* = −9.80 × 10^−4^ to −0.002, *p*_FDR_ = 4.97 × 10^−5^ to 0.02; Figure 4d,e). Among the specific mental and behavioral disorders, 10 significant BAG‐mediated associations between heart IDPs and diseases were identified, including substance abuse, mood disorders, and neurotic disorders (Table S9). For example, the largest mediation effect was observed for mood disorders, with BAG mediating 8.82% of the negative associations between RV IDPs and disease prevalence (indirect effect: *β* = −0.003, *p*_FDR_ = 1.07 × 10^−5^). In addition, BAG showed a significant mediating effect in the negative association between RV IDPs and the prevalence of substance abuse (proportion = 7.22%, indirect effect: *β* = −0.003, *p*_FDR_ = 1.16 × 10^−6^).

Similarly, BAG significantly mediated the three associations between two heart IDP categories (LV and RV) and the broad category of nervous system disorders (*p*_FDR_ < 0.05, Figure 4f–h), with BAG exhibiting the largest mediating effect in the negative association between RV IDPs and disease prevalence (proportion = 7.62%, indirect effect: *β* = −0.002, *p*_FDR_ = 3.95 × 10^−4^; Figure 4f). In addition, BAG also mediated both positive (proportion = 2.92%, indirect effect: *β* = 0.001, *p*_FDR_ = 0.002; Figure 4g) and negative (proportion = 4.83%, indirect effect: *β* = −8.90 × 10^−4^, *p*_FDR_ = 0.003; Figure 4h) associations between LV IDPs and the prevalence of nervous system disorders. Take together, these results indicate that the ventricle structure show stronger brain aging‐related mediation patterns in association with brain disorders, consistent with their central role in systemic circulation (Hill et al. 2024).

Sex‐stratified mediation analyses revealed that the direction of the mediation effects between heart IDPs, BAG, and brain disease outcomes was consistent across males and females, and aligned with the patterns observed in the combined‐sample analysis. No directionally opposite mediation effects were observed in either sex. These findings indicate that the mediating role of BAG is broadly similar across sexes. Detailed results of the sex‐stratified analyses are provided in Table S10. In addition, in sensitivity analyses restricted to incident disease outcomes, mediation effects were generally consistent in direction with those observed in the primary analyses using prevalent diseases (Table S11). Following hierarchical FDR correction, all 18 originally significant mediation effects retained statistical significance (Table S9). Furthermore, after excluding rare disorders (psychotic disorders and Parkinson's disease), the results remained highly consistent. All previously significant mediation pathways remained significant with consistent effect directions (Table S12), indicating that findings were not driven by low‐prevalence conditions.

### 2.5 Identifying Environmental and Lifestyle Factors for Heart‐Brain Aging‐Disorder Associations

**FIGURE 5:**
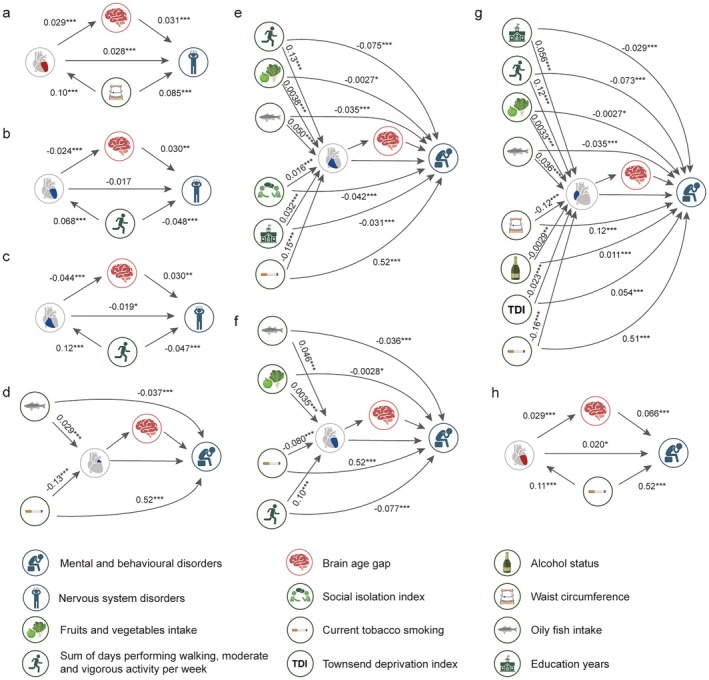
Mediation analysis shows lifestyle factors affect mental and nervous system diseases through heart IDP categories and BAG. Panels (a–h) illustrate significant pathways linking environmental and lifestyle factors, heart IDP categories (LV, RV, LA, RA), BAG, and mental and nervous system diseases. For each lifestyle factor and the mental and nervous system diseases, including nervous system disorders (Panels a–c) and mental and behavioral disorders (Panels d–h), separate structural equation model (SEM) was fitted to examine whether lifestyle factors influence these diseases through their effects on the heart and BAG. Significant paths are illustrated where heart IDP categories and BAG demonstrated significant mediating effects. Color‐coded heart IDPs are displayed at their respective anatomic locations. Since BAG consistently exhibited positive associations with brain diseases, the red color indicates the positive associations between heart IDPs and BAG or diseases, while blue color represents the negative associations between heart IDPs and BAG or diseases. All models were estimated using a 1000‐iteration non‐parametric bootstrap approach. *FDR‐corrected *p* < 0.05; **FDR‐corrected *p* < 0.01; ***FDR‐corrected *p* < 0.001. BAG, brain age gap; FDR, false discovery rate; IDPs, imaging‐derived phenotypes; LA, left atrium; LV, left ventricle; RA, right atrium; RV, right ventricle.

Additional SEM analyses were further conducted to investigate whether the potential modifiable environmental and lifestyle factors were associated with brain disorders through a serial mediation chain of heart IDPs and BAG (a × b × d, Figure 1). Several lifestyle factors were significantly associated with the broad category of nervous system disorders through LV IDPs and RV IDPs, and BAG (Figure 5a–c). Larger waist circumference was associated with higher LV IDPs and accelerated brain aging, as well as a higher prevalence of diseases. In contrast, higher levels of physical activity, such as sum of days performing walking and moderate and vigorous activity per week, were linked to larger LV IDPs and RV IDPs, delayed brain aging and lower prevalence of diseases (Figure 5a–c).

Similarly, we observed a total of 46 significant associations (*p*_FDR_ < 0.05), where lifestyle factors were significantly correlated with mental and behavioral disorders (both the broad category and specific disorders) via the potential mediating roles of four heart IDP categories (i.e., LV, LA, RA, and RV IDPs) and BAG (Figure 5d–h). Detailed statistics of associations are presented in Table S13. Overall, in line with the existing evidence (Tian et al. 2024), smoking status (e.g., current tobacco smoking), alcohol status, Townsend deprivation index, and waist circumference were associated with accelerated brain aging (a × d) and higher disease prevalence. Healthy diet (e.g., fruits and vegetables intake) and physical activity (e.g., sum of days performing walking, moderate and vigorous activity per week) were linked to delayed brain aging. Notably, current tobacco smoking and oily fish intake were the only two lifestyle factors that consistently associated with mental and behavioral disorders across all four heart IDP categories (Figure 5d–h and Table S13). In contrast, several lifestyle items exhibited more specific association patterns. For instance, social isolation index was linked to higher RV IDPs, reduced brain aging and lower disorders prelalence (Figure 5e), while waist circumference, alcohol status, and Townsend deprivation index were associated with lower RA IDPs, accelerated brain aging and elevated prevalence of disorders (Figure 5g).

After refinement of the SEM structure, including addition of a direct lifestyle‐to‐BAG path and exclusion of blood pressure and diabetes from the lifestyle construct, the mediation results remained largely consistent. Of the 49 previously significant mediation pairs, 35 remained significant in the revised model, and all showed consistent effect directions. These findings support the robustness of the proposed pathways (Table S14). In sensitivity analysis, 41 out of 49 significant associations remained significant after hierarchical FDR correction (Table S13).

## 3 Discussion

While the relationships of cardiac health with mental and nervous system disorders have been widely observed, the potential mediating role of brain aging in these associations remains underexplored. This study integrated heart and brain imaging data from the UKB to examine whether brain aging mediates the relationship between cardiac health and brain diseases. Using GLM regression model, we identified 61 heart IDPs that show distinct associations with brain diseases. Mediation analyses further revealed that brain aging significantly mediates many of these heart‐disease associations. Additionally, we identified modifiable environmental and lifestyle factors associated with heart IDPs and BAG, which were themselves linked to mental and nervous system disorders. Together, these findings suggest that brain aging may function as a potential mediator within the observed associations between cardiac function and mental and nervous disorders. By identifying specific cardiac phenotypes and lifestyle factors correlated with accelerated brain aging, our results highlight potential targets for early identification and integrated lifestyle‐heart‐brain interventions in the aging population.

One possible explanation for these findings is the hemodynamic hypothesis, which suggests that impaired cardiac function, such as reduced cardiac output or arrhythmias, can lead to cerebral hypoperfusion (de la Torre 2012; Gardarsdottir et al. 2018; Moroni et al. 2020; Moore and Jefferson 2021). Chronic cerebral hypoperfusion has been associated with neuroimaging markers of brain aging, including increased white matter lesions and accelerated atrophy (Moroni et al. 2020; Alosco et al. 2013; Schuff et al. 2009; Appelman et al. 2008; Ghaznawi et al. 2021). Previous evidence also suggests that cardiac traits such as the left ventricle are associated with brain atrophy (Park et al. 2017; Jefferson et al. 2010). Additionally, we found that LV myocardial mass and wall thickness showed stronger associations with BAG, which are consistent with prior research in which LV geometry appeared as one of the cardiac traits most strongly associated with hippocampal volume (Masci 2025), supporting the idea that impaired cardiac output may be associated with brain aging and increase the risk of mental and nervous system disorders.

Another possible explanation involves systemic neuroinflammatory and neuroendocrine mechanisms. Cardiac dysfunction, particularly in heart failure, has been associated with systemic inflammation (Yndestad et al. 2006; Murphy et al. 2020; Ridker and Lüscher 2014). Recent evidence suggests that systemic inflammatory signals may cross the blood–brain barrier and activate microglial cells, the brain's resident immune cells (Hoogland et al. 2015; Dionisio‐Santos et al. 2019; Chouhan et al. 2022). Microglial activation has been implicated in synaptic loss, white matter damage, and impaired neurogenesis—hallmarks of accelerated brain aging (Dionisio‐Santos et al. 2019; Li et al. 2023). These processes may, in turn, increase the vulnerability to multiple mental and nervous system disorders, underscoring the mediating role of brain aging in our study.

Moreover, dysregulation of the autonomic nervous system may represent another possible linkage between heart IDPs and brain aging. The heart and brain are known to be interconnected through sympathetic and parasympathetic pathways (Hu et al. 2023). Heart structural remodeling, such as myocardial hypertrophy or fibrosis, has been associated with altered afferent signaling to brainstem cardiovascular control centres (e.g., the nucleus tractus solitarius (Ziegler et al. 2025)), which in turn has been linked to central autonomic dysfunction (Tahsili‐Fahadan and Geocadin 2017; Toyli et al. 2025). Since the central autonomic network is responsible for maintaining the cerebral blood flow (Zhang et al. 2002), such autonomic dysfunction has been correlated with reduced cerebral blood flow (Toyli et al. 2025; Mankoo et al. 2023), which has itself been associated with accelerated aging (Tarumi and Zhang 2018; Juttukonda et al. 2021; Beishon et al. 2022). Together, these observations provide a physiologically plausible explanation for the associations between cardiac features and global brain aging observed in our study.

Our findings build on and extend prior research linking cardiac structure to mental and nervous system disorders (Zhao et al. 2023; Bai et al. 2020). First, we confirmed previously reported associations between heart IDPs, particularly RA and RV IDPs, and mood disorders and neurodegenerative conditions (Bai et al. 2020). Notably, a recent large‐scale genetic study based on UKB data identified associations between multiple LV IDPs (i.e., ejection fraction, wall thickness, and myocardial strain) and both schizophrenia and bipolar disorder (Zhao et al. 2023). While this study emphasizes shared genetic architecture between cardiac and disease phenotypes, our findings extend this evidence by revealing the associations at the structural imaging level. Second, we identified novel associations, such as between LV end‐diastolic volume and dementia, likely enabled by the greater statistical power from our larger sample size compared to prior studies (*N* for this study = 33,573 vs. *N* for prior research = 26,893). Furthermore, our study also extends the scope to include a broader range of mental and nervous system disorders, including substance abuse, neurotic disorders, epilepsy, and sleep disorders. Our findings reinforce the hypothesis that cardiac health is linked to a wide range of mental and nervous system disorders (Shen et al. 2022; Zhao et al. 2023; Jefferson et al. 2010; Bai et al. 2020; Qiu and Fratiglioni 2015). Finally, while prior studies have largely examined direct associations between cardiac phenotypes and clinical disease outcomes, our findings suggest that brain aging may mediate the relationships between heart traits to mental and nervous system disorders, offering a novel insight to the heart‐brain axis framework.

Furthermore, we also identified multiple modifiable environment and lifestyle factors that may contribute to the observed heart‐brain aging‐disorder pathways. Smoking, physical activity, and diet style were all significantly linked to both cardiovascular and brain health (Mintzer et al. 2019; Dong et al. 2025). In particular, smoking status was associated with all four heart IDP categories (i.e., LV, RV, LA, and RA), which in turn were linked to brain aging and disease outcomes. These findings are in line with prior evidence linking smoking to adverse cardiac remodeling (Fried et al. 2022) (e.g., left ventricular hypertrophy (Kamimura et al. 2018), reduced end‐diastolic volume, and impaired right ventricular function (Schafnitzel et al. 2019)), increased arterial stiffness (Hahad et al. 2023), and systemic inflammation (Shiels et al. 2014; Çolak et al. 2019). These alterations may impair cardiac output and cerebral perfusion (Tarumi et al. 2011; Winder et al. 2021) and accelerate brain aging (Moore and Jefferson 2021; Dionisio‐Santos et al. 2019; Tsao et al. 2016). Together, these associations provide a potential mechanistic pathway from lifestyle factors to mental and nervous system disorders, with brain aging acting as a mediator between cardiac traits and neuropsychiatric outcomes.

Despite these contributions, several limitations warrant discussion. First, the UKB cohort is not population‐representative, as participants are predominantly of white European ancestry and higher socioeconomic status, which may limit the generalizability of our findings. Future studies should aim to include more diverse ethnic and socioeconomic backgrounds to improve external validity. Second, several limitations related to potential confounding should be acknowledged. Certain unmeasured factors, such as hypertension treatments, lipids, and genetic factors, may be directly correlated with brain aging and even mental and nervous system disorders, which could affect the interpretation of the observed heart IDPs‐BAG‐disorders associations. For instance, antihypertensive medications, such as angiotensin‐converting enzyme inhibitors and angiotensin‐II receptor blockers, have been shown to be related to both cardiac structural changes and brain health outcomes with some evidence suggesting that direct neuroprotective properties independent of blood pressure control (Santiago et al. 2023; Gray et al. 2026). Similarly, although the extended SEM framework allowed us to simultaneously examine direct relationships between lifestyles and disorders, as well as indirect potential pathways mediated by heart IDPs and BAG, some of these unmeasured factors may also simultaneously relate to both heart and brain, further affecting the interpretation of the observed potential pathways. Taken together, while residual confounding may exist to some extent, the consistency of findings across multiple sensitivity analyses suggests that the observed associations are unlikely to be entirely attributable to such factors. Furthermore, the cross‐sectional design and limited longitudinal data restrict our ability to draw causal inferences. As our findings were particularly derived from mediation analyses based on observational data, they reflect associational relationships rather than evidence of a causal role for BAG between heart traits and mental and nervous system disorders. Future longitudinal studies will be essential for clarifying the causality of these associations.

In conclusion, this study provides important evidence that brain aging may serve as a potential mediating role linking cardiac structure and function to a wide range of mental and nervous system disorders. By integrating heart and brain imaging data with disease phenotypes and lifestyle exposures, we identified specific cardiac IDPs associated with brain disease outcomes, with BAG significantly mediating these associations. These findings support the conceptualization of brain aging as a plausible pathway within the heart–brain axis and underscore the relevance of cardiovascular health in understanding individual differences in brain vulnerability. From a translational perspective, these results underscore the potential value of cardiovascular screening and lifestyle interventions in promoting both cardiac and brain health. Tracking changes in heart IDPs over time may offer a non‐invasive approach for identifying individuals at elevated risk for mental and nervous system disorders and may facilitate early targeted interventions that integrate cardiovascular and neurological care in the aging population.

## 4 Methods

### 4.1 Study Population

Heart and brain MRI‐derived phenotypes were obtained from the UKB, a large‐scale biomedical database containing genetic, lifestyle and health data from over 500,000 UK participants aged 40 to 96 (Sudlow et al. 2015; Bycroft et al. 2018). The UKB received approval from the North West Multi‐center Research Ethics Committee, and all participants in the UKB have provided written informed consent.

This study analyzed a subset of participants who underwent extensive physical and physiological assessments and completed questionnaires on environmental and lifestyle factors. After excluding the participants missing more than 20% of features, a total of 33,573 participants remained (mean age, 63.5 years; SD, 7.5; 53.3% women; 92.6% white). Among them, 20,964 individuals (10,388 males) did not have a clinical diagnosis of mental and behavioral disorders (ICD‐10 F00‐F99) or nervous system disorders (ICD‐10 G00‐G99). 12,609 (5290 males) individuals had a lifetime diagnosis of one of the following mental and nervous system disorders, including mental and behavioral disorders (*N* = 8874), dementia (*N* = 152), substance abuse (*N* = 2310), psychotic disorders (*N* = 66), mood disorders (*N* = 4582), neurotic disorders (*N* = 4206), nervous system disorders (*N* = 8394), Parkinson's disease (*N* = 110), epilepsy (*N* = 347), transient ischemic attack (*N* = 567), and sleep disorder (*N* = 1145).

### 4.2 MRI‐Derived Heart and Brain Phenotypes

IDPs of heart and brain were obtained using multisequence MRI. For the heart, 82 IDPs related to cardiac and aortic structure and function (Bai et al. 2020) were analyzed using R software (Table S15). According to physiological structures and functions, these IDPs were divided into 6 categories: LV (64 IDPs), RV (4 IDPs), LA (4 IDPs), RA (4 IDPs), Aao (3IDPs) and Dao (3 IDPs) (Zhao et al. 2023).

For the brain, 1453 IDPs were derived from T1‐weighted and diffusion‐weighted MRI scans, comprising 1010 gray matter features and 443 white matter features (Table S16). Gray matter features included regional volume, cortical thickness, and surface area (*n* = 1010), while white matter features encompassed mean values of fractional anisotropy, diffusivity, intracellular volume fraction, isotropic volume fraction, water molecule diffusion eigenvalues, orientation dispersion, mode of anisotropy, and white matter volume (*n* = 443). Brain IDPs were generated using the centrally implemented and standardized UKB image‐processing pipeline, which integrates publicly available imaging tools from the FMRIB Softwar Library (FSL, version 5.0.10) and FreeSurfer (version 6.0) (https://biobank.ctsu.ox.ac.uk/crystal/crystal/docs/brain_mri.pdf). All the methods for quality control imaging processing have been extensively validated previously (Alfaro‐Almagro et al. 2018).

### 4.3 Mental and Nervous System Diseases

In total, 11 mental and nervous system disorders were analyzed in UKB (Table S1), which were divided into two broad categories (mental and behavioral disorders and nervous system disorders), with the remaining nine being non‐exhaustive representative diseases from these two categories. From the first broad category, mental and behavioral disorders, five disorders were further analyzed: dementia (ICD‐10 F00‐F03, G30, G31), substance abuse (ICD‐10 F10‐F19), psychotic disorders (ICD‐10 F20‐F29), mood disorders (ICD‐10 F30‐F39), and neurotic disorders (ICD‐10 F40‐F48). From the second broad category, nervous system disorders (ICD‐10 G00‐G99), four disorders were analyzed: Parkinson's disease (ICD‐10 G20‐G22), epilepsy (ICD‐10 G40‐G42), transient ischemic attacks (ICD‐10 G45‐G46), and sleep disorders (ICD‐10 G47). Diagnoses were based on hospital records, primary care data, self‐reports, and death records, and censored at the last recorded follow‐up date (You et al. 2023).

### 4.4 Environmental and Lifestyle Factors

Following prior research on modifiable factors in UKB studies (Tian et al. 2024) and cardiovascular disease risk factors (Yusuf et al. 2020), we included 14 lifestyle and environmental exposures. These factors were categorized into nine groups, including physical activity, sleep quality, diet, smoking status, alcohol consumption, social isolation index, education level, socioeconomic status, and abdominal obesity.

1. Physical activity: total number of days per week spent walking and engaging in moderate or vigorous physical activity (Field ID: 22033).
2. Sleep quality: frequency of insomnia/sleeplessness (Field ID: 1200, 1, never/rarely; 2, sometimes; 3, usually).
3. Diet: assessed separately for oily fish intake (Field ID: 1329, 0, never; 1, less than once a week; 2, once a week; 3, two to four times a week; 4, five or six times a week; 5, once or more daily), processed meat intake (Field ID: 1349, 0, never; 1, less than once a week; 2, once a week; 3, two to four times a week; 4, five or six times a week; 5, once or more daily), salt added to food (Field ID: 1478, 1, never/rarely; 2, sometimes; 3, usually; 4, always), and overall fruit and vegetable consumption (per day), including the sum of fresh fruit (Field ID: 1309), dried fruit (Field ID: 1319), salad/raw (Field ID: 1299), and cooked vegetables (Field ID: 1289) intake.
4. Smoking status: current tobacco smoking (Field ID: 1239, which was re‐coded to: 0 = No; 1 = only occasionally; 2 = Yes, on most or all days, so that higher scores denoted higher frequency of current smoking), exposure to tobacco smoke at home (Field ID: 1269), and exposure to tobacco smoke outside home (Field ID: 1279).
5. Alcohol consumption: total weekly red wine intake (Field ID: 1568), average weekly champagne plus white wine intake (Field ID: 1578), average weekly beer plus cider intake (Field ID: 1588), average weekly spirits intake (Field ID: 1598), and average weekly fortified wine intake (Field ID: 1608). The alcohol consumption of individuals who indicated that they never drink (Field ID: 20117) was set to zero.
6. Social isolation index: derived from household size (Field ID: 709, one point assigned for living alone). Frequency of social visits (Field ID: 1031, responses of “once a month,” “once a few months,” “never,” or “almost never” were recoded, with higher scores indicating more frequent visits).
7. Education level: Age at which full‐time education was completed (Field ID: 845).
8. Social economic status: assessed using the Townsend deprivation index (Field ID: 22189).
9. Abdominal obesity: waist circumference (Field ID: 48).

### 4.5 Statistical Analysis

#### 4.5.1 Data Preprocess

The missing data were imputed using *K*‐nearest neighbor (KNN, *n*_neighbors = 50) imputation. All heart and brain IDPs were normalized using the mean and SD across all individuals. The effects of scanning centres and total intracranial volume were removed from brain IDPs before calculating the BAG, while heart IDPs were deconfounded for scanning centres and BMI before subsequent analyses.

#### 4.5.2 Brain Biological Age Prediction Model

To estimate brain biological age, we constructed a chronological age prediction model using brain IDPs via LASSO regression (alpha = 1) using the R package GLMnet v4.1.8. The model was trained on healthy participants (*N* = 4936, mean age, 63.8 years, 51.8% women) with no lifetime medical records in linked primary care, hospital inpatient data, death registers, or self‐reports (Category 1712). It was then applied to the remaining UKB participants (*N* = 28,637). The biological age was defined as the outcome and brain IDPs were used as the predictors. In the training process, a 10‐fold cross‐validation strategy was used to prevent overfitting. Following the 10‐fold cross‐validation procedure, model performance was evaluated in each iteration using the MAE on the held‐out fold. The model achieving the lowest MAE across the test folds was selected as the final model and used for all subsequent analyses. Within the training data in each outer cross‐validation fold, the optimal regularization parameter (*λ*) was selected using 10‐fold internal cross‐validation across a predefined grid of *λ* values (10^5^ to 10^−8^) (Liu et al. 2025). The *λ* value minimizing MAE was selected. No additional retraining on the full healthy sample was performed after cross‐validation (Tian et al. 2023). Specifically, in each iteration of the model training, nine of the folds were used to determine the optimal model, with the effects of the scanning site and total intracranial volume regressed from the IDPs, and all variables standardized. Specifically, all variables were standardized using *Z*‐score normalization (mean = 0, SD = 1), with the scaling parameters estimated from the training folds and subsequently applied to the corresponding test fold. The fitted covariates regression model was also applied to the remaining fold, after which the optimal model was applied to predict the brain biological age. For overall performance visualization, predictions from all held‐out folds were pooled to compute a single correlation coefficient and MAE between predicted brain biological age and chronological age. Within each fold, model performance was also evaluated separately and summarized as mean ± SD across folds.

Predicted brain age is known to exhibit age‐dependent bias, typically manifesting as overestimation in younger individuals and underestimation in older individuals. To address this, we applied a bias‐correction procedure following the approach recommended by Smith et al. (2019). Within each cross‐validation fold, predicted age was regressed on chronological age using the training data. The resulting intercept (*β*_0_) and slope (*β*_1_) were then applied to the corresponding test data to obtain bias‐corrected predicted age:

$$\mathrm{Age}\_\text{corrected}=\left(\mathrm{Age}\_\text{predicted}-\beta_{0}\right)/\beta_{1}$$

The BAG was calculated as the difference between bias‐corrected predicted age and chronological age, where a positive value (BAG > 0) indicated accelerated aging and a negative value (BAG < 0) indicated delayed aging, providing insights into brain health. Importantly, correction parameters were derived exclusively from training data to prevent data leakage.

In addition, we performed additional sensitivity analyses using SVR and RF. Both alternative models were trained and evaluated using the same 10‐fold cross‐validation scheme as the primary LASSO model, with identical preprocessing steps applied within each fold. Model performance was assessed on the held‐out fold in each cross‐validation iteration using the same performance metrics.

#### 4.5.3 Generalized Linear Model

A GLM was conducted using the R‐implemented “glm” function to examine the association between heart IDPs and BAG, adjusting for chronological age, sex, and scanning site. Significant associations were determined with a FDR‐corrected two‐sided *p*‐value < 0.05.

For diagnosis before imaging (mean sample size = 31,150, mean cases = 308, Table S5), GLM logistic regression was used to assess the associations between heart IDPs and selected diseases. For diagnosis occurring after imaging (sample size = 33,637, mean cases = 2487, Table S6), the associations between heart IDPs and risk of diseases were evaluated by Cox proportional hazards models (R package survival v3.6.4). All heart IDPs were standardized using *Z*‐score normalization before entering two models. Thus, the reported HRs reflect the relative risk associated with a one SD increase in each heart IDP. Furthermore, to facilitate clinical interpretation, the SD of each heart IDP was additionally calculated based on raw data. Both models were adjusted for chronological age, sex, smoking and drinking status, BMI, and Townsend index. Statistical significance was set at FDR‐corrected *p* < 0.05.

A GLM logistic regression model was also constructed to assess the relationship between BAG and the eleven selected diseases. If BAG was positively correlated with a disease, the directional effects among heart IDPs, BAG, and disease risk were expected to align; otherwise, they were expected to be inverse. Associations meeting these criteria were used in next SEM analysis. The remaining associations were categorized by heart IDP type. Mean values for each heart IDP subcategory were computed and incorporated into the SEM analysis.

#### 4.5.4 Mediation Analysis

SEM was employed to assess whether BAG mediates the association between heart IDPs and mental and nervous system diseases. Coefficients a, b and c represent the effects of heart IDPs on BAG, BAG on disease outcomes and the total effect, respectively. The direct effect (*c′*) quantifies changes in disease outcomes when heart IDPs increase by one unit and the mediator remains unchanged. SEM was initially employed to assess the significance of pathways (a × b) from mean heart IDPs to mental and nervous system diseases, with the BAG as the mediator. Heart IDPs were included in the mediation analysis only if they were significantly associated with both BAG and diseases. Separate SEM models were developed for each category of heart IDPs and each of the eleven selected diseases. The proportion of mediation was determined by calculating the ratio (a × b)/c. Statistical significance was determined using a threshold of FDR‐corrected *p* < 0.05 across all examined models. SEM models were retained if the paths of a, a × b, and c were statistically significant. These models were further expanded to include lifestyle factors. All models were adjusted for chronological age and sex. Model coefficients and significance levels were estimated using the R package Lavaan (v0.6.19).

In sensitivity analysis, to examine whether the mediating role of BAG was consistent across sexes, additional sex‐stratified mediation analyses were conducted. Specifically, separate structural equation models were fitted in males and females, with chronological age included as a covariate. In addition, to address concerns regarding temporal ordering in the mediation analyses, a sensitivity analysis was conducted using incident disease outcomes only. Incident cases were defined as diagnoses occurring after the imaging visit.

Consistent with prior research, the performance of the model fit of each SEM model was evaluated using three indices: the comparative fit index (CFI), the root mean square error of approximation (RMSEA), and the standardized root mean square residual (SRMR). A model was considered satisfactory if CFI > 0.90 or RMSEA < 0.05 or SRMR < 0.08 (Tian et al. 2024).

For those pathways identified as statistically significant in the original mediation analysis, further SEM analysis was conducted to evaluate the significance of pathways (a × b × d) from lifestyle factors (exogenous variable) to mental and nervous system diseases (outcome), with mean heart IDPs and the BAG acting as mediators. The coefficients a, b, and c retained the same interpretation as in SEM models without lifestyle factors. Coefficient *d* represented the effect of lifestyle on heart IDPs, while *e* captured the total effect of lifestyle factors on disease outcomes. Separate SEM models were constructed for each lifestyle factor, with statistical significance determined at FDR‐corrected *p* < 0.05 across all examined models. In response to concerns regarding potential bidirectional relationships between certain cardio‐metabolic variables and cardiac structure, we refined the extended SEM specification. Specifically, a direct path from lifestyle factors to BAG was added to allow for independent effects of lifestyle on brain aging. The revised SEM was re‐estimated to evaluate the robustness of mediation effects under this alternative structural specification.

#### 4.5.5 Multiple Testing Correction

To account for the large number of statistical tests across multiple analytical layers, following the framework proposed by Gillis et al. (2025), we further conducted sensitivity analyses by implementing a hierarchical FDR correction procedure across all major analyses. Specifically, FDR correction was first applied across families to control for global multiplicity and subsequently within each family to identify significant associations while maintaining overall error control.

## Author Contributions

W.C. proposed and administrated the study. W.C., Y.J., and P.R. designed the study. Z.Z., W.S., P.R., and Y.J. analyzed the data. W.C., Y.J., P.R., and W.S. contributed to the interpretation of results. Z.Z., W.S., P.R., and Y.J. drafted the manuscript. W.C., Y.L., B.Z., F.L., and D.L. edited the manuscript. W.S., P.R., and Y.J. contributed to the visualization. W.C., J.‐T.Y., and J.F. were responsible for funding support. All authors read and approved the final paper.

## Funding

W.C. was supported by grants from the Noncommunicable Chronic Diseases—National Science and Technology Major Project (2025ZD0546300), the National Key R&D Program of China (No. 2023YFC3605400), the National Natural Science Foundation of China (Nos. 82472055, 62433008), the Shanghai Pilot Program for Basic Research—Fudan University 21TQ1400100 (25TQ010), and Shanghai Science and Technology Commission Program (23JS1410100). J.‐T.Y., as SANS Scholar, was supported by grants from the Brain Science and Brain‐like Intelligence Technology—National Science and Technology Major Project (2022ZD0211600), National Natural Science Foundation of China (82530047, 82588301, 82271471), Leading Project of the Discipline Breakthrough Plan of the Ministry of Education (JYB2025XDXM604), Excellent Academic Research Leader Program of Shanghai (23XD1420400), Changping Laboratory (2025B‐07‐35), Lin Gang Laboratory (LGL‐3241‐PDA030200), Shanghai Academy of Natural Sciences, New Cornerstone Science Foundation, Zhangjiang Lab, Tianqiao and Chrissy Chen Institute, and the State Key Laboratory of Brain Function and Disorders of Ministry of Education, Fudan University. J.F. was supported by the 111 Project (No. B18015), the Shanghai Center for Brain Science and Brain‐Inspired Technology and the Zhangjiang Lab. Peng Ren was funded by China Postdoctoral Science Foundation (2025M772197). The funders had no role in study design, data collection and analysis, decision to publish or preparation of the manuscript.

## Ethics Statement

The UKB received approval from the North West Multi‐center Research Ethics Committee.

## Consent

All participants in the UKB have provided written informed consent.

## Conflicts of Interest

The authors declare no conflicts of interest.

## Supporting information


**Figure S1:** Age distribution of participants included in the brain imaging analyses. The histogram shows the frequency of individuals at each chronological age. Age 70 years represents the most frequent age in the cohort and was therefore selected for age‐specific analyses of inter‐individual variability in brain IDPs.
**Figure S2:** Distributions of representative brain IDPs among individuals aged 70 years. Six brain IDPs were randomly selected from the full set of IDPs. Histograms illustrate substantial inter‐individual variability in brain structural measures despite identical chronological age. Mean values and standard deviations are shown for each IDP.
**Figure S3:** Unsupervised clustering of brain IDPs in age‐specific groups. K‐means clustering was performed separately for individuals aged 70 years (top row) and for individuals aged 66–70 years (bottom row). The optimal number of clusters was determined using the elbow method (left panels). Clustering results were visualized using principal component analysis (PCA) for dimensionality reduction (right panels), illustrating latent structure within same‐age and narrow age‐range populations.
**Figure S4:** Association between chronological age and brain age gap (BAG) before and after bias correction. (A) Panels (a) and (b) show results in healthy individuals used for model training and cross‐validation; panels (c) and (d) show results in the full sample. (a, c) Before correction, BAG exhibited substantial negative correlation with chronological age (*r* = −0.55), reflecting age‐related prediction bias. (b, d) After correction using parameters derived from the training data, the association between BAG and chronological age was markedly attenuated (healthy sample: *r* = −0.07; full sample: *r* = −0.05), indicating effective removal of age‐related bias.

**Table S1:** Definition and sample size of mental and nervous system disorders.
**Table S2:** Fold‐wise performance of the brain age prediction model in 10‐fold cross‐validation.
**Table S3:** Performance of Support Vector Regression (SVR) and Random Forests (RF) models in 10‐fold cross‐validation.
**Table S4:** Association between heart IDPs and brain age gap (BAG).
**Table S5:** Association between BAG and prevalence of mental and nervous system disorders.
**Table S6:** Association between BAG and incidence of mental and nervous system disorders.
**Table S7:** Sensitivity analysis of logistic regression models after exclusion of rare disorders.
**Table S8:** Sensitivity analysis of Cox proportional hazards models after exclusion of rare disorders.
**Table S9:** Mediation analysis of BAG in the relationship between heart IDPs and mental and nervous system disorders.
**Table S10:** Sex‐stratified mediation analyses of BAG in the associations between cardiac IDPs and brain disease outcomes.
**Table S11:** Mediation analyses between cardiac IDPs, brain age gap, and incident disease outcomes.
**Table S12:** Sensitivity analysis of mediation models after exclusion of rare disorders.
**Table S13:** Collective influence of lifestyles, heart and brain on mental and nervous system disorders for average heart IDPs.
**Table S14:** Mediation results from the revised SEM including a direct lifestyle‐to‐BAG path.
**Table S15:** The description of heart imaging‐derived phenotypes (IDPs).
**Table S16:** The description of brain IDPs.

## Data Availability

The individual level data analyzed in this study are available from the UK Biobank (https://biobank.ctsu.ox.ac.uk) upon application and approval. The code for the main analysis of this study is publicly available at https://github.com/WenJSu/Heart‐brain‐aging‐disorders‐pathways.
