# Supplementary material for: Brain Aging Mediating Heart Imaging‐Derived Phenotypes and Mental and Nervous System Disorders
Source: Aging Cell. 2026 Apr 23;25(5):e70499. doi: 10.1111/acel.70499 (PMC13103654; doi:10.1111/acel.70499)
Supplement: Supplementary file 1 — Figure S1: Age distribution of participants included in the brain imaging analyses. The histogram shows the frequency of individuals at each chronological age. Age 70 years represents the most frequent age in the cohort and was therefore selected for age‐specific analyses of inter‐individual variability in brain IDPs. Figure S2: Distributions of representative brain IDPs among individuals aged 70 years. Six brain IDPs were randomly selected from the full set of IDPs. Histograms illustrate substantial inter‐individual variability in brain structural measures despite identical chronological age. Mean values and standard deviations are shown for each IDP. Figure S3: Unsupervised clustering of brain IDPs in age‐specific groups. K‐means clustering was performed separately for individuals aged 70 years (top row) and for individuals aged 66–70 years (bottom row). The optimal number of clusters was determined using the elbow method (left panels). Clustering results were visualized using principal component analysis (PCA) for dimensionality reduction (right panels), illustrating latent structure within same‐age and narrow age‐range populations. Figure S4: Association between chronological age and brain age gap (BAG) before and after bias correction. (A) Panels (a) and (b) show results in healthy individuals used for model training and cross‐validation; panels (c) and (d) show results in the full sample. (a, c) Before correction, BAG exhibited substantial negative correlation with chronological age (r = −0.55), reflecting age‐related prediction bias. (b, d) After correction using parameters derived from the training data, the association between BAG and chronological age was markedly attenuated (healthy sample: r = −0.07; full sample: r = −0.05), indicating effective removal of age‐related bias. [file ACEL-25-e70499-s002.docx]

**Brain aging mediating heart imaging-derived phenotypes and mental and nervous system disorders**

# Supplementary Methods

# Supplementary Figures

**Figure S1** Age distribution of participants included in the brain imaging analyses.

**Figure S2** Distributions of representative brain IDPs among individuals aged 70 years.

**Figure S3** Unsupervised clustering of brain IDPs in age-specific groups visualized.

**Figure S4** Association between chronological age and brain age gap (BAG) before and after bias correction

**Supplementary Methods**

**Age-specific heterogeneity and clustering analyses**

To assess inter-individual variability in brain imaging–derived phenotypes (IDPs) among individuals of the same chronological age, the age distribution of the imaging cohort was first examined. We identified the most frequent age and selected the age group for age-specific analyses.

For descriptive purposes, six brain IDPs were randomly selected from the full set of 1,453 IDPs, and their distributions among the participants in the most frequent age were visualized using histograms. Mean values and standard deviations were calculated to illustrate variability within this age group.

To explore potential latent structure within same-age and narrow age-range populations, unsupervised clustering analyses were conducted using K-means clustering. Analyses were performed separately for individuals in the most frequent age group and for those in the five most frequent age groups. The optimal number of clusters was determined using the elbow method based on within-cluster sum of squares. Principal component analysis (PCA) was subsequently applied for dimensionality reduction and visualization of clustering results.

# Supplementary Figures


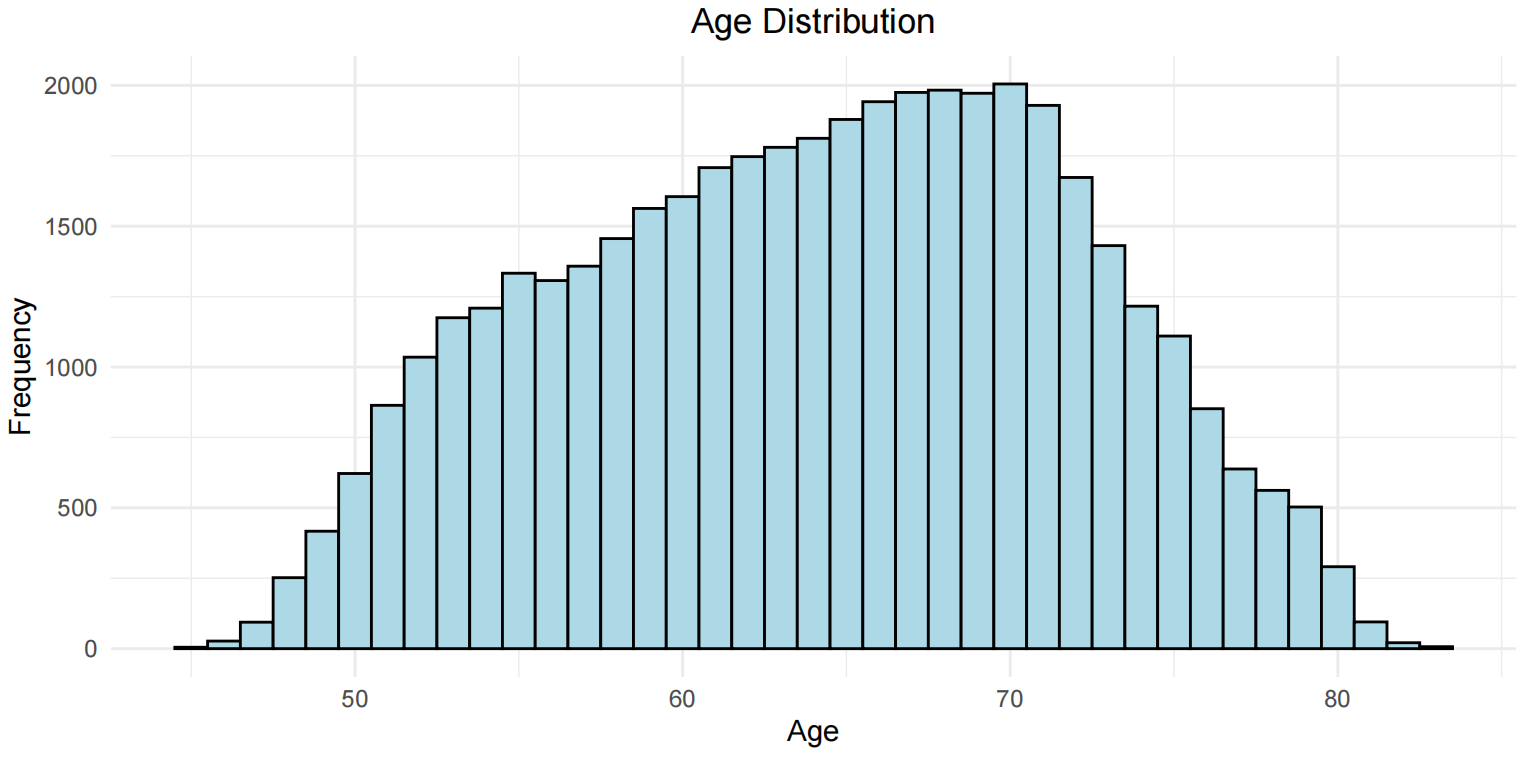


**Figure S1 Age distribution of participants included in the brain imaging analyses.** The histogram shows the frequency of individuals at each chronological age. Age 70 years represents the most frequent age in the cohort and was therefore selected for age-specific analyses of inter-individual variability in brain IDPs.


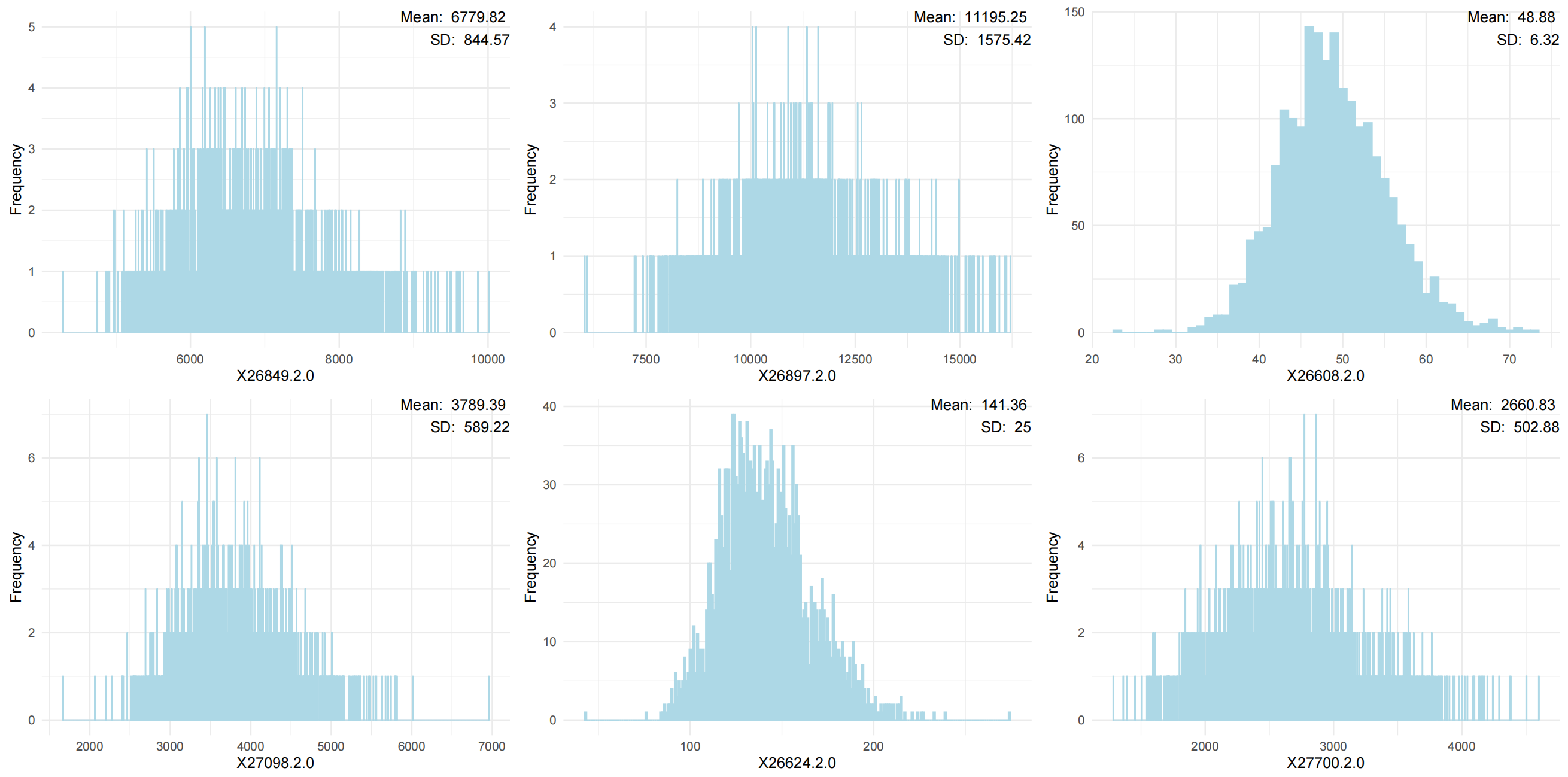


**Figure S2 Distributions of representative brain IDPs among individuals aged 70 years.** Six brain IDPs were randomly selected from the full set of IDPs. Histograms illustrate substantial inter-individual variability in brain structural measures despite identical chronological age. Mean values and standard deviations are shown for each IDP.


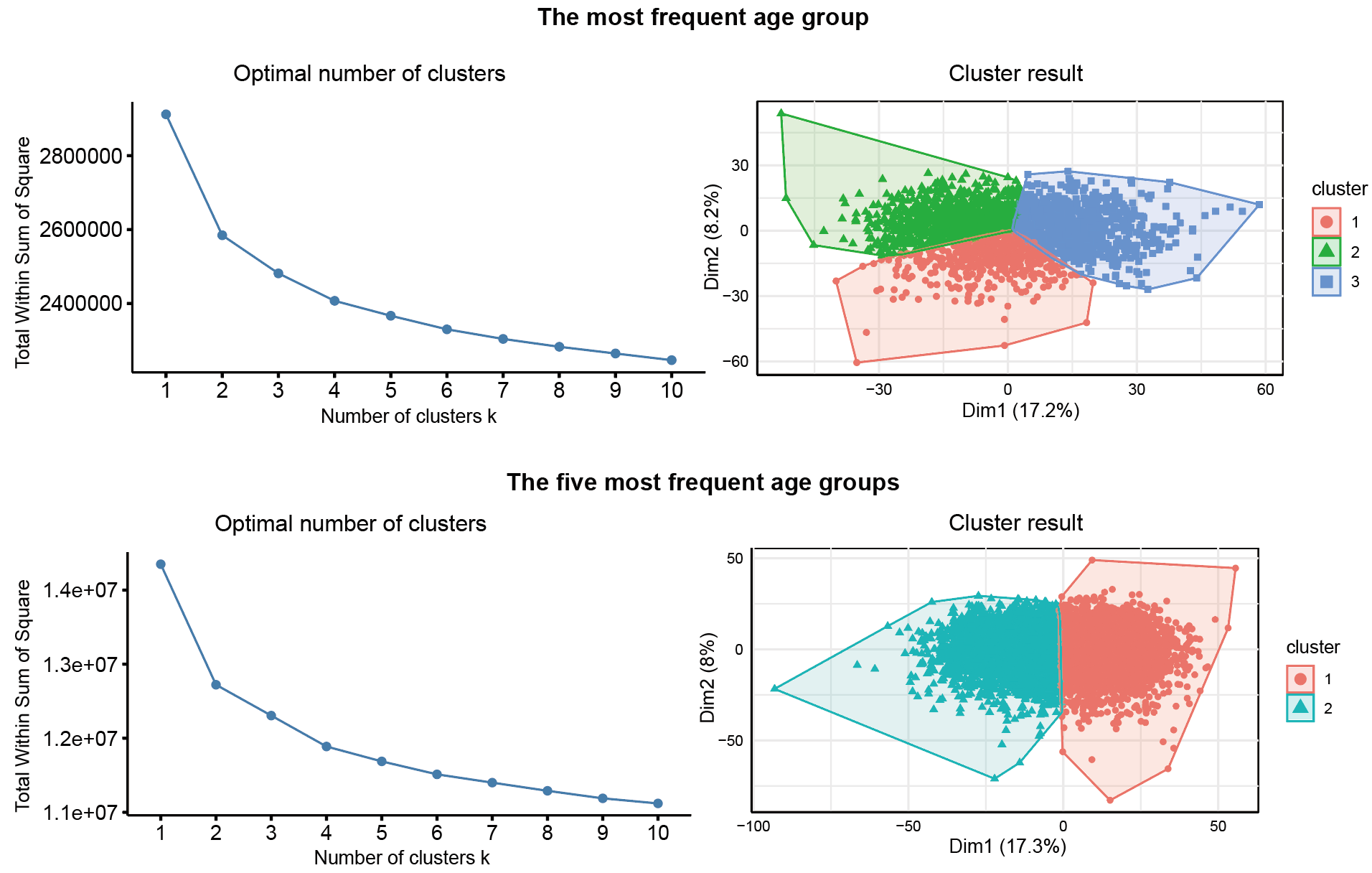


**Figure S3 Unsupervised clustering of brain IDPs in age-specific groups.** K-means clustering was performed separately for individuals aged 70 years (top row) and for individuals aged 66–70 years (bottom row). The optimal number of clusters was determined using the elbow method (left panels). Clustering results were visualized using principal component analysis (PCA) for dimensionality reduction (right panels), illustrating latent structure within same-age and narrow age-range populations.


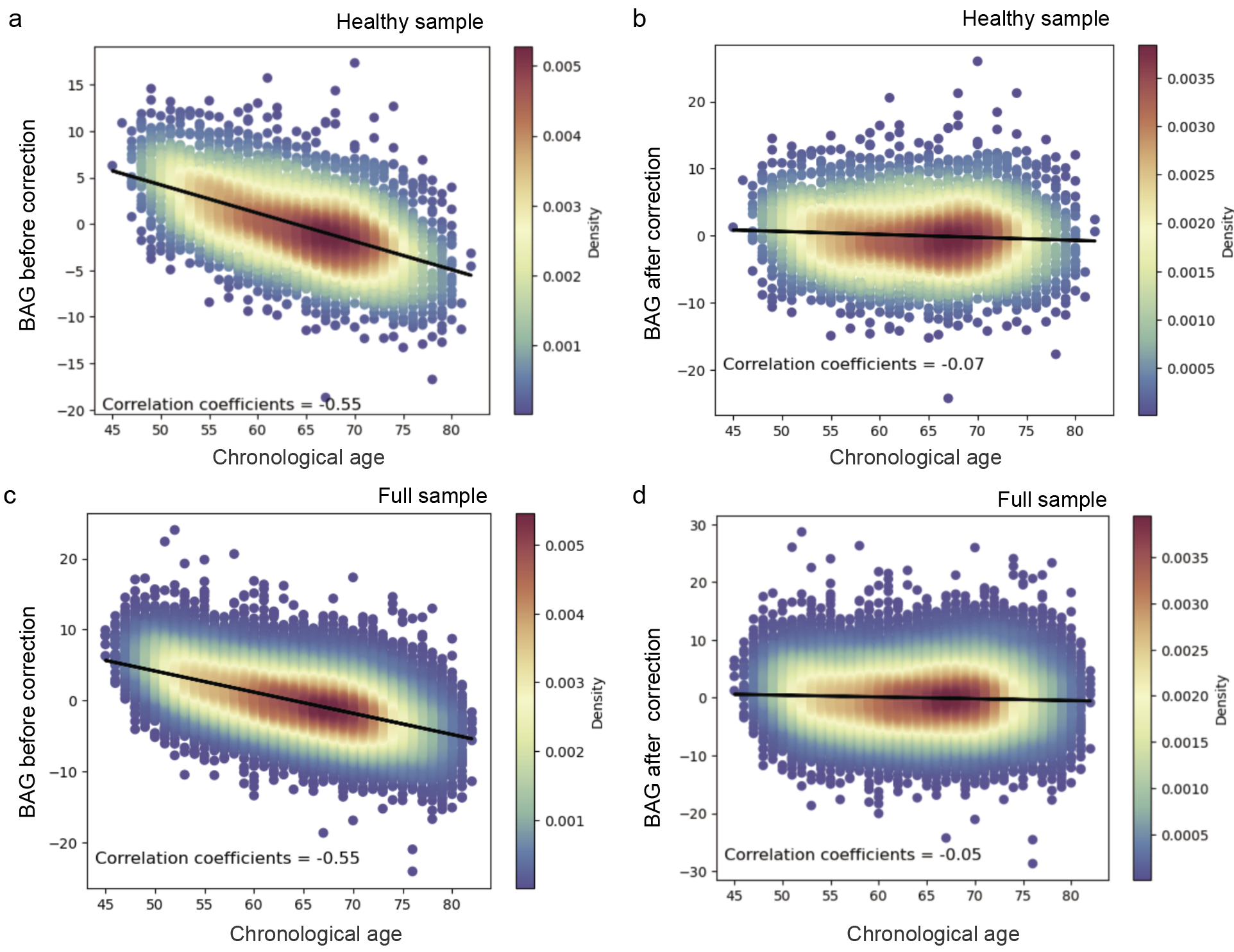


**Figure S4 Association between chronological age and brain age gap (BAG) before and after bias correction.** (A) Panels (a) and (b) show results in healthy individuals used for model training and cross-validation; panels (c) and (d) show results in the full sample. (a, c) Before correction, BAG exhibited substantial negative correlation with chronological age (r = −0.55), reflecting age-related prediction bias. (b, d) After correction using parameters derived from the training data, the association between BAG and chronological age was markedly attenuated (healthy sample: r = −0.07; full sample: r = −0.05), indicating effective removal of age-related bias.
